# An Improved Method for Cell Type-Selective Glycomic Analysis of Tissue Sections Assisted by Fluorescence Laser Microdissection

**DOI:** 10.3390/ijms20030700

**Published:** 2019-02-06

**Authors:** Chiaki Nagai-Okatani, Misugi Nagai, Takashi Sato, Atsushi Kuno

**Affiliations:** Glycoscience and Glycotechnology Research Group, Biotechnology Research Institute for Drug Discovery, National Institute of Advanced Industrial Science and Technology (AIST), Tsukuba, Ibaraki 305-8568, Japan; chiaki-okatani@aist.go.jp (C.N.-O.); nagai.misugi@aist.go.jp (M.N.); takashi-sato@aist.go.jp (T.S.)

**Keywords:** glycomic profiling, tissue section, immunohistochemistry, laser microdissection, lectin microarray

## Abstract

Lectin microarray (LMA) is a highly sensitive technology used to obtain the global glycomic profiles of endogenous glycoproteins in biological samples including formalin-fixed paraffin-embedded tissue sections. Here, we describe an effective method for cell type-selective glycomic profiling of tissue fragments collected by laser microdissection (LMD) under fluorescent histochemical visualization. We optimized each step of histochemical staining and confirmed the reliability and validity of glycomic profiling. Using the optimized procedure, glycomic profiles were obtained with 0.5 mm^2^ of stained thymic sections (5-μm-thick) from 8-week-old C57BL/6J male mice. The glycomic profiles of *Ulex europaeus* agglutinin-I (UEA-I)-stained medullary regions showed higher UEA-I signals than those of the morphologically determined medulla regions, indicating the utility of this method for UEA-I(+) cell-selective analysis. To further evaluate this method, tissue fragments was serially collected from stained and unstained areas of medullary epithelial cell probes (UEA-I and anti-cytokeratin 5 antibody) and a cortex-staining probe (peanut agglutinin). The medullary regions assigned by the three probes showed significantly different glycomic profiles, highlighting the difference in subpopulation recognition among the three probes, which was consistent with previous reports. In conclusion, our fluorescence LMD-LMA method enabled cell type-selective tissue glycomic analysis of pathological specimens and animal models, especially for glyco-biomarker discovery.

## 1. Introduction

As one of the most important posttranslational modifications, protein glycosylation has crucial biological and physiological roles, from contributions to protein folding and quality control to involvement in various types of biological recognition events [1]. Disease-related alterations in protein glycosylation are promising targets for the discovery of glyco-biomarkers and development of therapeutic agents [2,3]. Lectin microarray (LMA) is a lectin-assisted glycomic technology used to obtain glycomic profiles of *N*- and *O*-glycans in glycoprotein samples without glycan liberation steps, enabling evaluation of the characteristics of glycan structures using simple and rapid procedures [4,5]. In LMA, glycoprotein samples are fluorescently labeled at their primary amine groups, allowing for glycans attached to glycoproteins to be selectively analyzed [5]. Thus, LMA facilitates mass spectrometry-based glycoproteomics for detailed analyses of glycosylation sites and their glycan structures [6,7], and hence this technology is useful for the most upper phases of glyco-biomarker discovery [3,8]. LMA is applicable for glycomic profiling formalin-fixed paraffin-embedded (FFPE) tissue specimens [9], which are a highly valuable source for many disease-related studies. Using this technique, disease-related glycosylation alterations have been detected in various types of cancer [9,10,11,12]. Recently, we standardized the LMA analysis procedure of FFPE tissue sections through the combined use of laser microdissection (LMD), which allowed for precise collection of populations of cells from specific microscopic regions of tissue sections by direct visualization [13]; this LMD-LMA method has been applied to obtain comprehensive glycome mapping data of FFPE tissue sections to overlook site- and tissue-specific protein glycosylation [14].

Here, for cell type-selective glycomic analysis, we aimed to develop an advanced procedure for LMD-LMA method, which directly analyzed glycomic profiles of fluorescently stained histochemical sections. To prove the concept, we used thymic sections of normal mice as representative analytes. The medulla and cortex contain different cell types and thus can be distinguished not only morphologically by their cellular density but also histochemically using lectins and antibodies against cell type-specific molecular markers [15,16]. Among these probes, we used the following three probes: the fucose-binding lectin *Ulex europaeus* agglutinin-I (UEA-I) that recognizes a carbohydrate epitope expressed in a distinct subset of medullary thymic epithelial cells (mTECs) [17]; peanut agglutinin (PNA), a lectin staining immature cortical thymocytes but not mature medullary thymocytes [18]; and an antibody against cytokeratin 5 (CK5), which is expressed in a subpopulation of mTECs [19].

## 2. Results

### 2.1. Optimization of Sample Preparation for Specific Probe-Stained Sections

**Figure 1:**
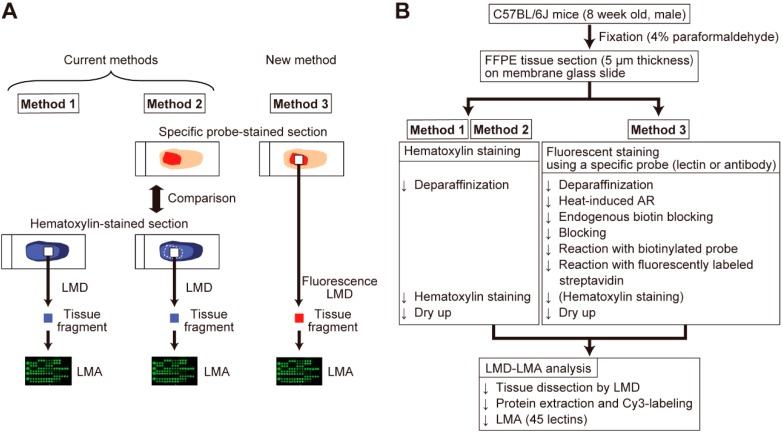
Comparison of the current and new methods for laser microdissection–lectin microarray (LMD-LMA) analysis. (**A**) Comparison of tissue dissection. In Method 1, tissue fragments were collected from hematoxylin-stained formalin-fixed paraffin-embedded (FFPE) tissue sections based on morphological observation by LMD. For cell type-selective collection, Method 2 employed two serial sections, where one was stained with a cell type-specific probe for observation and one with hematoxylin for tissue dissection by LMD. In contrast to these current methods, in the new method called Method 3, tissue fragments were collected directly from the section that was fluorescently stained with a cell type-specific probe under fluorescent observation. This fluorescence LMD allowed for a more accurate and reproducible tissue collection. The procedure after the tissue dissection for LMA analysis was the same in these three methods. (**B**) Schematic overview of LMD-LMA analysis of hematoxylin- and fluorescent-stained tissue sections.

For cell type-selective tissue analysis, tissue sections stained with a cell type-specific probe were essentially prepared for assignment of dissected areas. As shown in Figure 1A, the present strategy called fluorescence LMD-LMA (Method 3) only utilized a single tissue section that was fluorescently stained with a specific probe, whereas Method 2 involved tissue dissection from a hematoxylin-stained section while referring to another probe-stained section. Thus, we first optimized a new procedure for tissue section preparation suitable for the fluorescence LMD-LMA using 45 lectins (Table S1), as summarized in Figure 1B. In this procedure, a biotin-streptavidin detection system was employed to ensure the versatility of the probes. To enhance laser absorption efficiency, additional hematoxylin staining can be performed if this step does not affect the fluorescent staining pattern. In the course of optimization, we compared two membrane glass slides (i.e., polyethylene naphthalate (PEN) and polyphenylene sulfide (PPS)) used for LMD-LMA analysis of UEA-I-stained thymic sections and found that similar glycomic profiles were obtained with these membrane glass slides (Figure S1 and Table S2).

### 2.2. Effects of Fluorescent Staining Procedures on Glycomic Profiling

Because most commercialized lectins and antibodies are glycoproteins, we examined whether these probes remained in the protein extracts prepared from fluorescently stained tissue fragments and affected their glycomic profiles. As a representative glycosylated probe, we estimated the amount of the remaining biotinylated UEA-I contaminating the protein extracts of the tissue fragment obtained from the UEA-I(+) regions of UEA-I-stained sections. By using a western blot-like chemiluminescence detection system, the remaining probe amount was estimated to be below 62.5 pg in 0.5 mm^2^ tissue fragment-derived extracts (Figure S2A). We also found that biotinylation of the probe caused significant reduction in its Cy3-labeling efficiency due to its masking effect on the primary amine groups of the probe (Figure S2B), and hence the biotinylated probe showed a much lower signal than a non-labeled probe in LMA (Figure S2D). Additionally, western blot analysis using Cy3-labeled probes and tissue extracts demonstrated that contamination by the staining probe was sufficiently low and could be ignored in the glycomic profiling (Figure S2C,E). Similarly, we also evaluated the amount and effects of contaminated anti-CK5 antibody in CK5(+) tissue samples, obtaining similar results as was the case with UEA-I (Figure S3).

To further evaluate the effects of the remaining probes on glycomic profiling, we compared the glycomic profiles of the same areas obtained from three serial thymic sections separately stained with the following three methods: (1) hematoxylin (corresponding to the current procedure), (2) hematoxylin following antigen retrieval (AR), and (3) UEA-I and hematoxylin following AR (corresponding to the new procedure) (Figure S4A–C). The protein amount obtained from the tissue fragments with AR was decreased to one-third of that of untreated tissue fragments (Figure S4D); however, tissue fragments of as small as 0.5 mm^2^ could be used to obtain glycomic profiles even with AR (Table S3). By adjusting the amount of the protein extracts used for analysis, the UEA-I-stained sample group showed no significant difference in the relative signal intensities of the 45 lectins, compared to hematoxylin-stained sections with AR (Student’s *t*-test, *p* > 0.05; Figure S4E). These results indicate that the staining procedure did not affect the resultant glycomic profiles.

Collectively, the present results indicate that fluorescence LMD-LMA can be applied for differential glycomic profiling.

### 2.3. Comparison of Differential Glycomic Profiles Obtained by the Current and New Methods

**Figure 2:**
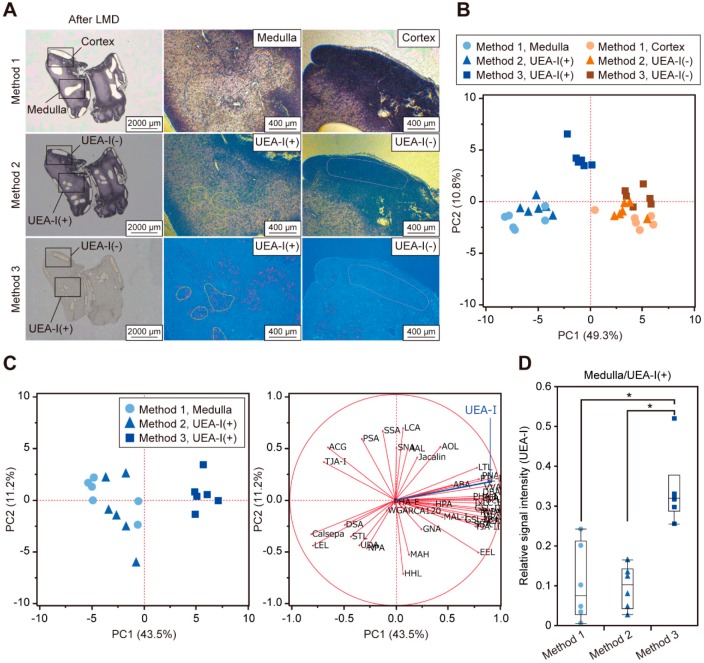
Differential glycomic profiling of tissue fragments obtained from medulla/*Ulex europaeus* agglutinin-I (UEA-I)(+) and cortex/UEA-I(−) regions by each method. Serial thymic sections were stained with hematoxylin or with UEA-I, and then subjected to LMD-LMA analysis by Methods 1, 2, and 3. (**A**) Whole section images after dissection (left panels) and representative images of tissue dissection at the areas indicated in the left panels (middle and right panels). Whole section images before or after tissue dissection with sample annotations are shown in Figure S5A. (**B**) Principle component analysis (PCA) score plot of glycomic profiles of tissue fragments obtained from the two regions by each method. (**C**) PCA score plot (left panel) and loading plot (right panel) of glycomic profiles of the medulla/UEA-I(+) tissue fragments. In the loading plot, the vector corresponding to UEA-I is highlighted in blue. (**D**) Relative UEA-I signal intensities of the medulla/UEA-I(+) tissue fragments. Asterisks indicate significant differences between groups (Tukey’s honestly significant difference, *p* < 0.05). Glycomic profile data (0.5 mm^2^ equivalent; *n* = 6 each) used for these analyses are presented in Table S4.

Next, to evaluate the utility of combining histochemical staining with tissue glycomic profiling, we compared the glycomic profiles of the tissue fragments obtained from three serial thymic sections by Methods 1, 2, and 3. In Method 1, the medullary and cortex regions were separately collected based on the degree of hematoxylin staining; in contrast, the UEA-I(+) and UEA-I(−) regions were dissected from the hematoxylin- and UEA-I-stained sections in Methods 2 and 3, respectively (Figure 2A and Figure S5A,B). In the score plot of principle component analysis (PCA), the resultant glycomic profiles (Table S4) formed distinct clusters, according to the medulla/UEA-I(+) and cortex/UEA-I(−) regions analyzed by each method (Figure 2B). Consistent with these results, the statistically significant differences in the glycomic profiles of the two regions were observed for each method (Table S4). The coefficient of variation values of the relative signal intensities of the 45 lectins in the samples (*n* = 6 for each group) was similar among the cortex/UEA-I(−) groups; in contrast, among the medulla/UEA-I(+) groups, the UEA-I(+) samples prepared by Method 3 showed a relatively smaller value. Additionally, among the medulla/UEA-I(+) samples of these three methods, samples analyzed by Method 3 showed different glycomic profiles with relatively higher UEA-I signals (Figure 2C,D). Taken together, we found that Method 3 provided reproducible and specific results in LMD-LMA analysis for probe-stained cells.

### 2.4. Differential Glycomic Profiling Using Histochemical Sections Stained with Different Probes

**Figure 3:**
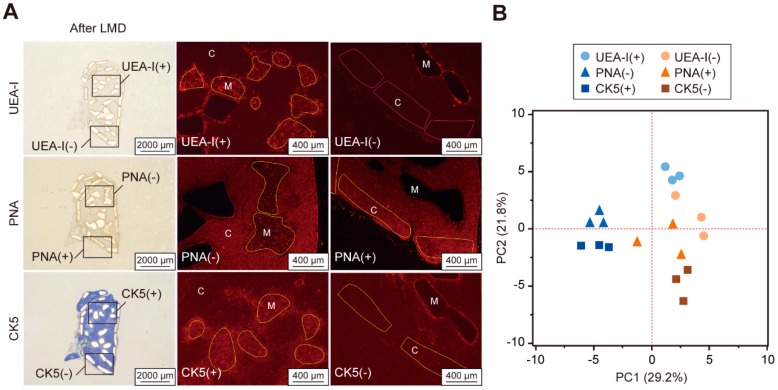
Differential glycomic profiling of tissue fragments obtained from stained and unstained regions with the three probes. Serial thymic sections were stained for UEA-I, peanut agglutinin (PNA), and cytokeratin 5 (CK5) and then subjected to LMD-LMA analysis by Method 3. (**A**) Whole section images after dissection (left panels) and representative fluorescent images of tissue dissection from the probe-stained and unstained regions indicated in left panels (middle and right panels). Note that UEA-I and CK5 staining was observed predominantly in the medulla (*M*), whereas PNA staining was relatively higher in the cortex (*C*). The whole section images before or after tissue dissection with sample annotations are shown in Figure S6B. (**B**) PCA score plot of glycomic profiles of the tissue fragments obtained from the two regions of the three probe-stained sections. Glycomic profile data (0.5 mm^2^ equivalent; *n* = 3 each) used for these analyses are presented in Table S5.

As the reliability and utility of fluorescence LMD-LMA analysis has been demonstrated, we next applied this method to compare the glycomic profiles obtained with three serial thymic sections separately stained for UEA-I, PNA, and CK5. As probes for mTEC cells, UEA-I and an anti-CK5 antibody staining was observed predominantly in the medulla, whereas PNA staining was observed selectively in the cortex (Figure S6A). The stained- and unstained regions of these three probes were successfully dissected under fluorescent microscopy (Figure 3A and Figure S6B). In the LMA analysis, all the samples with the three probes yielded a similar mean signal intensity under the same scanning condition, indicating that the protein amount of these samples were similar to each other. We compared the resulting glycomic profiles (Table S5) by PCA, in which the samples of the medullary and cortex regions form different groups for all probes, highlighting the differences in the glycomic profiles (Figure 3B). For each probe, the signals of lectins recognizing α2,6-linked sialic acid (*Sambucus nigra* agglutinin (SNA), *Sambucus sieboldiana* agglutinin (SSA), and *Trichosanthes japonica* agglutinin-I (TJA-I)) were predominant in the cortex rather than in the medulla (Figure S6D–F). In contrast, several lectins showed different profiles between these two regions; relatively higher signals for high mannose-binders (*Narcissus pseudonarcissus* agglutinin (NPA) and *Galanthus nivalis* agglutinin (GNA)) were observed in the cortex compared to in the medulla by PNA or CK5 staining, but not by UEA-I staining. In addition, the signal of an *O*-glycan binder (soybean agglutinin (SBA)) was relatively higher in the cortex for the PNA or CK5 staining, but predominant in the medulla for the UEA-I staining.

Since diverse glycomic profiles were observed within the medullary groups of the three methods (Figure 3B), we further compared only samples from the medullary groups by PCA. The UEA-I(+) samples showed distinct glycomic profiles from samples in the other two groups (Figure S6C; left panel). The lectins responsible for the difference were identified from the corresponding PCA loading plot (Figure S6C; right panel) and statistical analysis of the relative signal intensities of the 45 lectins (Table S6). The signals of fucose binders (*Aleuria aurantia* lectin (AAL) and *Aspergillus oryzae lectin* (AOL)) were relatively lower in the UEA-I(+) samples, while multiple asialo *O*-glycan binders (PNA, *Dolichos biflorus* agglutinin (DBA), SBA, *Vicia villosa* agglutinin (VVA), and *Wisteria floribunda* agglutinin (WFA)) showed significantly higher signals in the UEA-I(+) group.

**Figure 4:**
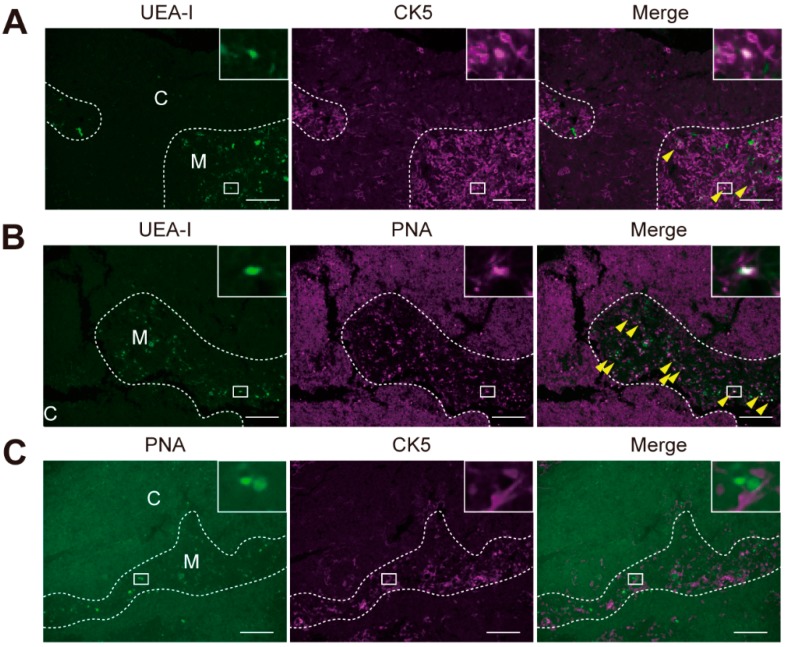
Representative images of double fluorescent staining for UEA-I and CK5 (**A**), UEA-I and PNA (**B**), and PNA and CK5 (**C**). Arrowheads indicate double-positive cells shown as white in merged images. Dashed lines indicate the border between the medulla (*M*) and cortex (*C*). Insets show enlarged images of the indicated areas. Scale bars, 100 μm.

To evaluate whether these characteristics in the glycomic profiles reflected differences in the dissected areas determined by the staining pattern, we compared the locations of each stained area by fluorescent double-staining. Staining of UEA-I and CK5 overlapped in part of the cell population located in the medulla (Figure 4A). In the medulla, PNA intensely stained part of the cell population, with many cells also stained with UEA-I (Figure 4B) but not with anti-CK5 antibody (Figure 4C). These double-staining patterns suggested that UEA-I(+) regions contained higher numbers of PNA ligand-expressing cells than CK5(+) regions, which was consistent with the PCA results of LMD-LMA analysis of the medullary regions (Figure S6C). Using a similar strategy, we compared the locations of the staining for UEA-I, PNA, and CK5 with the staining of another asialo *O*-glycan binder, DBA, which showed a higher signal in UEA-I(+) samples (Figure S6C and Table S6). We found that DBA staining in the medulla overlapped with UEA-I or PNA staining, but not with staining for CK5 (Figure S7). This was consistent with the observation that UEA-I(+) regions showed higher DBA signals than CK5(+) regions. Based on these results, fluorescence LMD-LMA analysis with an appropriate probe (i.e., Method 3) revealed more cell type-selective glycomic profiles compared to conventional methods (i.e., Method 1 and Method 2).

## 3. Discussion

The previously developed LMD-LMA method is a powerful tool for detecting tissue- and site-specific protein glycosylation in FFPE tissue sections [14]; however, this method is not highly cell-type specific. The usefulness of histochemistry-based cell type-specific isolation of nucleic acids has been demonstrated in previous studies [20,21]. Here, by employing this strategy for tissue dissection procedures, we established an advanced procedure using fluorescently stained sections. In fact, the fluorescence LMD-LMA method provided reliable glycomic profiles reflecting cell type-selective protein glycosylation, which was not possible using the current methods.

The present glycomic profiles obtained from the medullary and cortex regions determined by the staining pattern of UEA-I, PNA, and CK5 clearly revealed the characteristics of protein glycosylations of these two regions. For all probes, higher signals in the medullary samples were observed for three lectins recognizing α2,6-linked sialic acids (SNA, SSA, TJA-I) compared with those in the cortical samples, whereas the situation was not for a lectin bound to α2,3-linked sialic acids (*Maackia amurensis* lectin-I (MAL-I)). These results were consistent with those of a previous report by Baum et al., which showed that SNA bound only to medullary mature thymocytes, whereas MAL-I bound to all thymocytes [22]. In contrast, the signals of several lectins, including MAL-I, *Lycopersicon esculentum* lectin (LEL), *Pisum sativum* agglutinin (PSA), *Datura stramonium* agglutinin (DSA), Concanavalin A (ConA), and *Helix pomatia* agglutinin (HPA), were not significantly different between the medullary and cortex regions. These results agreed with those of a previous report by Paessens et al. showing that histochemical staining was observed in both regions of human thymic sections [23]. This study also showed that the staining of two lectins for high mannose-type *N*-glycans (NPA and GNA) was observed in the cortex and Hassall’s corpuscles in the medulla [23]. Accordingly, the present glycomic profiles obtained using PNA- and CK5-stained sections showed relatively higher signals for NPA and GNA in the cortex regions. Such differences were not observed with UEA-I-stained sections, which was also reasonable because both UEA-I(+) and UEA-I(−) regions include carbohydrate epitopes of NPA and GNA [23]. Totally, the present glycomic profile data of the medullary and cortex regions were consistent with those of previous reports on the expression patterns of lectin ligands, indicating the reliability of fluorescence LMD-LMA analysis.

The present study also clearly showed differences in glycomic profiles between the medullary regions determined by UEA-I staining and those by staining for PNA and CK5. These results are reasonable because the dissected UEA-I(+) regions were in limited areas of the medulla compared to the CK5(+) and PNA(−) regions [24]. Because mTECs are roughly divided into UEA-I(+)CK5(−) and UEA-I(−)CK5(+) subpopulations [19,24], the differences in glycomic profiles between these probes highlight the difference in their recognition of mTECs subpopulations. Consistent with the double-staining analysis results, the UEA-I(+) samples showed relatively higher signals for multiple asialo *O*-glycan binders, including DBA and PNA. Although DBA was shown to bind to immature CD4(−)CD8(−) thymocytes located in the cortex [25], the medullary cell types stained with DBA have not been clarified. Based on the previous histochemical analysis of human thymic sections [23], co-staining of UEA-I and PNA observed in the present study occurred in thymic blood vessels and capillaries, as well as in Hassall’s corpuscles. Considering that the staining of DBA and PNA partly overlapped in the medulla, the glycomic profiles of UEA-I(+) strongly reflected protein glycosylation of Hassall’s corpuscles and medullary blood vessels. In this context, the present glycomic profiling data reflected protein glycosylations of different cell types based on the probe used, demonstrating the usefulness of the fluorescence LMD-LMA method.

As demonstrated using the two probes (i.e., UEA-I and anti-CK5 antibody) that recognize discrete mTEC subpopulations, the present fluorescent LMD-LMA method enabled differential glycomic analysis of cell subpopulations. An application example for the present method would be glycomic analysis on cancer stem cells (CSCs), which are a small subset of cells within a tumor that bear stem cell features and are considered a pivotal target for the eradication of cancer [26,27]. Since CSCs share most of their cell surface markers with stem cells, it is necessary to identify novel CSC markers that are not present in the bulk of the tumor or in other stem cells for selective detection and targeting of CSCs. CSCs have been classified into subpopulations with discrete natures, which can be discriminated by the expression and glycosylation state of specific cell surface markers [28,29]. In this context, the present glycomic profiling method would facilitate to clarify the differences in glycomes, as well as glycosylation states of specific markers, between CSCs and non-CSCs and between CSC subpopulations, using specific antibodies that discriminate these cells. The identification and characterization of glycan structures on CSCs and specific CSC markers would greatly assist in the discovery of novel markers and therapeutic targets.

Despite its reliability and utility, the present fluorescence LMD-LMA method has several limitations. This lectin-assisted glycomic profiling cannot provide detailed glycan structural information, thus, determination of such glycan analysis requires mass spectrometry-based approaches. Additionally, because the present cell isolation strategy based on the staining pattern cannot entirely exclude heterogeneity of the cell types, more detailed cell type-specific analysis requires other separation methods, such as flow cytometry.

In conclusion, our fluorescence LMD-LMA method enabled cell type-selective glycomic analysis of FFPE tissue sections with more accuracy and precision compared to previous methods. This histochemical-based cell type-selective collection strategy could be applied not only to crude glycoprotein samples but also to purified glycoproteins from FFPE sections of various tissues by immunoprecipitation techniques [30] in combination with antibody-overlay LMA analysis [31]. We expect that the present method could be applied to differential glycomic analysis of pathological specimens in combination with immunohistochemistry for disease-relevant markers and thus can contribute to the discovery of disease-related glyco-biomarkers and therapeutic targets.

## 4. Materials and Methods

### 4.1. Animals

All animal experiments were performed in accordance with relevant guidelines and regulations and approved by the Committee for the Experiments involving Animals at AIST (project code, 2018-082; date of approval, 06/07/2018). C57BL/6J mice were bred and housed in a specific pathogen-free animal facility and had free access to food and water.

### 4.2. Tissue Section Preparation

Eight-week-old male mice were anesthetized by intraperitoneal injection of pentobarbital (50 mg/kg body weight) and perfused from the left ventricle with 0.1 M phosphate-buffered saline (PBS), pH 7.4 for 10 min and then with 4% paraformaldehyde in PBS for 10 min. Thymic tissues collected from the mice were fixed with 4% paraformaldehyde in PBS for 24 h at room temperature (RT) and then embedded in paraffin using a tissue processor (TP1020; Leica Microsystems, Wetzlar, Germany) under the following conditions: 70% (*v*/*v*) ethanol; 80% ethanol; 90% ethanol; three washes with 100% ethanol; three washes with xylene; and embedding twice in paraffin wax for 1 h each time. The FFPE blocks prepared using a paraffin-embedding device (EG1160; Leica Microsystems) were sectioned on a sliding microtome (SM2000R; Leica Microsystems) equipped with disposable blades (S35; Feather Safety Razor, Osaka, Japan). For LMD, 5-μm-thick tissue sections were mounted on poly-l-lysine-coated PEN- and PPS-membrane glass slides (Leica Microsystems). For immuno- and lectin histochemistry, 2-μm-thick sections were placed onto Matsunami Adhesive Slide (MAS)-coated glass slides (Matsunami, Osaka, Japan). The sections were dried overnight at 42 °C and stored at RT until use.

### 4.3. Tissue Section Staining

All tissue sections were deparaffinized three times with xylene for 5 min each and rehydrated in graded concentrations of ethanol. Fluorescent histochemistry was performed using a biotin-streptavidin system, as described previously with some modifications [14]. Briefly, AR was achieved by autoclaving the samples in citrate buffer (pH 6.0; Agilent Technologies, Santa Clara, CA, USA) for 10 min at 110 °C. All washing steps were conducted three times with 10 mM PBS, pH 7.4. The sections were blocked with the streptavidin-biotin blocking kit (Vector Laboratories, Burlingame, CA, USA) according to the manufacturer’s instructions, and further blocked with carbo-free blocking solution (Vector Laboratories) for 1 h at RT. The sections for LMD were incubated overnight at 4 °C with biotinylated lectin (5 μg/mL in PBS; UEA-I and PNA; J-Oil Mills, Tokyo, Japan) or rabbit polyclonal anti-CK5 antibody (5 μg/mL in PBS; 905504; Biolegend, San Diego, CA, USA) that had been biotinylated using the biotin-labeling kit-NH_2_ (Dojindo, Kumamoto, Japan). The sections for LMD were further incubated with Alexa Fluor 594-conjugated streptavidin (1 μg/mL in PBS; Invitrogen, Carlsbad, CA, USA). For hematoxylin staining before LMD, the sections were washed with water and stained with Mayer’s Hematoxylin Solution (Wako, Osaka, Japan). After all staining processes, the sections were washed with Milli-Q water, air-dried at RT, and then subjected to tissue dissection within one day. For fluorescent double staining, after blocking as described above, the sections were serially incubated with a biotinylated probe (5 μg/mL in PBS; PNA, anti-CK5 antibody, and DBA from Vector Laboratories) for 5 h at RT, a fluorescent isothiocyanate-labeled lectin (5 μg/mL in PBS; UEA-I, PNA, DBA from Vector Laboratories) overnight at 4 °C, and Alexa Fluor 647-conjugated streptavidin (1 μg/mL in PBS; Invitrogen), and then mounted in ProLong Gold antifade reagent (Invitrogen). Images of the stained sections were obtained using a fluorescent microscope (BZ-X710; Keyence, Osaka, Japan).

### 4.4. Tissue Dissection and Protein Extraction

Tissue dissection using an LMD system (LMD7000) attached to a microscope (DM6000B) equipped with a triple-band pass filter (Leica Microsystems) and subsequent protein extraction were carried out, as described previously with some modifications [9,14]. Briefly, tissue fragments (total area of 0.5–1 mm^2^) were collected from hematoxylin- and fluorescently stained sections (5-μm-thick) under bright-field and fluorescent-optics, respectively, into a 0.5-mL tube (Axygen, Union City, CA, USA). After centrifugation at 20,000× *g* for 1 min at 4 °C, the fragments were heat-denatured in 200 μL of 10 mM citrate buffer, pH 6.0 for 1 h at 95 °C. After centrifugation at 20,000× *g* for 1 min at 4 °C, 4 μL of a 50% (*w*/*v*) slurry of microcrystalline cellulose (Sigma-Aldrich, St. Louis, MO, USA) in Dulbecco’s PBS (D-PBS; Takara Bio, Shiga, Japan) was added. After discarding 190 μL of the supernatant and adding 190 μL of D-PBS, the fragments were subjected to protein extraction with 20 μL of D-PBS containing 0.5% Nonidet P-40 by sonication (three times for 3 s each). After incubation for 1 h on ice and centrifugation at 20,000× *g* for 1 min at 4 °C, the supernatants were collected as protein extracts.

### 4.5. LMA Analysis

The LMA procedure for crude protein extracts was performed essentially as described previously [9,14]. Briefly, 20 μL of protein extracts were fluorescently labeled with Cy3-succinimidyl ester (10 μg protein equivalent; GE Healthcare, Buckinghamshire, UK) for 1 h at RT in the dark. After adjusting the volume to 60 μL with probing buffer (25 mM Tris-HCl, pH 7.5, containing 137 mM NaCl, 2.7 mM KCl, 500 mM glycine, 1 mM CaCl_2_, 1 mM MnCl_2_, and 1% Triton X-100), the sample solutions were incubated for 2 h at RT to quench the excess reagent. After appropriate dilution with the probing buffer if necessary, the sample solutions were added to a well (60 μL/well) on the lectin array chip (LecChip™; GlycoTechnica, Yokohama, Japan) containing triplicate spots of 45 lectins (Table S1). After incubation overnight at 20 °C and washing three times with probing buffer, the array chip was scanned using an evanescent-field fluorescence scanner (GlycoStation™ Reader 1200; GlycoTechnica). All data were analyzed using GlycoStation™ Tools Pro Suite 1.5 software (GlycoTechnica). The net intensity was calculated by subtracting the background from the signal intensity. The data under appropriate gain conditions providing the net intensities of below 40,000 for all spots were used to obtain the glycomic profiles, which were presented as the relative signal intensities of the 45 lectins normalized to the mean value of all lection signals.

### 4.6. Western Blot Analysis

Because total protein levels in the crude protein extracts prepared from tissue fragments were quite low for standard quantitation by colorimetric assays, protein levels were estimated by western blot analysis as follows: An aliquot of Cy3-labeled protein extracts prepared as described above was dissolved in sample buffer containing 100 mM dithiothreitol, heated for 5 min at 95 °C, separated by sodium dodecyl sulfate-polyacrylamide gel electrophoresis for 60 min at 30 mA/gel, and transferred onto polyvinylidene difluoride membranes (Bio-Rad, Hercules, CA, USA) for 60 min at 4 mA/cm^2^. After washing three times with Tris-buffered saline (TBS; 25 mM Tris-HCl, pH 7.5, containing 137 mM NaCl and 2.7 mM KCl), the membranes were blocked with 4% Block Ace (DS Pharma Biomedical, Osaka, Japan) for 1 h at RT. After washing three times with TBS containing 0.1% Tween 20 (TBS-T), the membranes were incubated with a mouse monoclonal anti-Cy3/Cy5 antibody (C0992; Sigma-Aldrich; used at a dilution of 1:3000 in TBS-T containing 0.4% Block Ace) for 50 min at 37 °C. After washing three times with TBS-T, the membranes were further incubated with peroxidase-conjugated goat anti-mouse IgG (115-035-003; Jackson ImmunoResearch, West Grove, PA, USA; used at a dilution of 1:20,000 in TBS-T containing 0.4% Block Ace) for 40 min at 37 °C. After washing three times with TBS-T and once with TBS, the chemiluminescence blot was analyzed with a membrane scanner (C-DiGit; LI-COR, Lincoln, NE, USA) using ImmunoStar LD (Wako). Densitometric analyses were performed using ImageJ software (available online: http://rsb.info.nih.gov/ij/; NIH, Bethesda, MD, USA). At least three independent experiments were performed.

The amount of biotinylated probe contaminating the crude samples was estimated using a western blot-like chemiluminescence detection system as follows: An aliquot of protein extracts prepared as described above and biotinylated probe (i.e., UEA-I or anti-CK5 antibody) were simultaneously treated as described above to obtain the transferred membranes. After washing three times with TBS, the membranes were blocked with carbo-free blocking solution for 1 h at RT and then incubated with peroxidase-conjugated streptavidin (016-030-084; Jackson ImmunoResearch; used at a dilution of 1:20,000 in carbo-free blocking solution) for 1 h at RT. The membranes were analyzed as described above. At least two independent experiments were performed.

### 4.7. Statistical Analysis

Statistical comparison was performed with Student’s unpaired *t*-test and Tukey’s honestly significant difference tests for two and three groups, respectively. The differences were considered significant when *p* < 0.05. Statistical analyses and PCA for multivariable data were performed using JMP software (version 13.2.1; SAS Institute, Cary, NC, USA).

## Supplementary Materials

Supplementary materials can be found at http://www.mdpi.com/1422-0067/20/3/700/s1. Figure S1: Differential glycomic profiling of tissue fragments obtained from UEA-I stained sections on PEN- and PPS-membrane glass slides. Figure S2: Evaluation of the effects of UEA-I on LMA analysis. Figure S3: Evaluation of the effects of anti-CK5 antibody on LMA analysis. Figure S4: Differential glycomic profiling of tissue fragments obtained from hematoxylin- and UEA-I-stained sections. Figure S5: Tissue sections used for differential glycomic profiling of tissue fragments obtained from medulla/UEA-I(+) and cortex/UEA-I(−) regions by each method. Figure S6: Differential glycomic profiling of tissue fragments obtained from stained and unstained regions with the three probes. Figure S7: Representative images of double fluorescent staining for UEA-I and DBA, DBA and CK5, and PNA and DBA. Table S1: Abbreviations and carbohydrate specificities of 45 lectins on LecChip. Table S2: Differential glycomic profiles of UEA-I-stained tissue fragments (0.5 mm^2^ equivalent each) on PEN- and PPS-membrane glass slides. Table S3: Differential glycomic profiles of tissue fragments (0.5 mm^2^ equivalent each) obtained from the hematoxylin- and UEA-I-stained sections. Table S4: Differential glycomic profiles of tissue fragments (0.5 mm^2^ equivalent each) obtained from the medulla/UEA-I(+) and cortex/UEA-I(−) regions by each method. Table S5: Differential glycomic profiles of tissue fragments (0.5 mm^2^ equivalent each) of stained and unstained regions with the three probes. Table S6: Summary of significant differences among the glycomic profiles of the medullary regions determined using the three probes.

## Abbreviations

AAL	*Aleuria aurantia* lectin
AOL	*Aspergillus oryzae* lectin
AR	antigen retrieval
CK5	cytokeratin 5
ConA	Concanavalin A
CSC	cancer stem cell
DBA	*Dolichos biflorus* agglutinin
DSA	*Datura stramonium* agglutinin
FFPE	formalin-fixed paraffin-embedded
GNA	*Galanthus nivalis* agglutinin
HPA	*Helix pomatia* agglutinin
LEL	*Lycopersicon esculentum* lectin
LMA	lectin microarray
LMD	laser microdissection
MAL-I	*Maackia amurensis* lectin-I
mTEC	medullary thymic epithelial cell
NPA	*Narcissus pseudonarcissus* agglutinin
PBS	phosphate-buffered saline
PCA	principle component analysis
PEN	polyethylene naphthalate
PNA	peanut agglutinin
PPS	polyphenylene sulfide
PSA	*Pisum sativum* agglutinin
RT	room temperature
SBA	soybean agglutinin
SNA	*Sambucus nigra* agglutinin
SSA	*Sambucus sieboldiana* agglutinin
TJA-I	*Trichosanthes japonica* agglutinin-I
UEA-I	*Ulex europaeus* agglutinin-I
VVA	*Vicia villosa* agglutinin
WFA	*Wisteria floribunda* agglutinin
