# Supplementary material for: An Improved Method for Cell Type-Selective Glycomic Analysis of Tissue Sections Assisted by Fluorescence Laser Microdissection
Source: Int J Mol Sci. 2019 Feb 6;20(3):700. doi: 10.3390/ijms20030700 (PMC6387264; doi:10.3390/ijms20030700)

An improved method for cell type-selective glycomic analysis of tissue sections assisted by fluorescence laser microdissection

Chiaki Nagai-Okatani, Misugi Nagai, Takashi Sato, and Atsushi Kuno

Supplementary Figures and Legends

Figure S1. Differential glycomic profiling of tissue fragments obtained from UEA-I stained sections on PEN- and PPS-membrane glass slides. Serial thymic sections were mounted on each membrane glass slide, fluorescently stained with UEA-I, and subjected to LMD-LMA analysis by Method 3. **(A)** Fluorescent images. Left panels are whole section images. Middle panels are enlarged images of the rectangle regions shown in the left panels. Right panels are enlarged images of the rectangle regions shown in the middle panels. **(B)** Overview of tissue dissection. Left panels show whole section images after dissection from UEA-I(+) and UEA-I(-) areas (0.5 mm^2 each), which were determined by UEA-I staining pattern as shown in right panels. The numbers of dissected areas correspond to those of the glycomic profile data presented in Table S2. **(C)** PCA score plot of the glycomic profiles of UEA-I(+) and UEA-I(-) tissue fragments obtained from each membrane glass slide (0.5 mm^2 equivalent; N = 3 each).

Figure S2. Evaluation of the effects of UEA-I on LMA analysis. (A) Detection of biotinylated UEA-I. Protein extracts prepared from UEA-I(+) tissue fragments (T; 0.5 mm^2 equivalent) by Method 3, and the indicated amount of biotinylated UEA-I were subjected to SDS-PAGE, transferred to the same membrane, and detected with horseradish peroxidase-conjugated streptavidin. The arrow indicates the bands corresponding to biotinylated UEA-I. **(B)** Comparison of Cy3-labeling efficiency among UEA-I probes with or without biotinylation and heating. Non-

labeled and biotinylated UEA-I were treated with or without heating with citrate buffer and then subjected to labeling with Cy3-SE. The Cy3-labeled UEA-I probes (1 ng each) were subjected to western blotting analysis using anti-Cy3 antibody. The arrow indicates the bands corresponding to biotinylated UEA-I. **(C)** Comparison of protein amounts among non-labeled and biotinylated UEA-I after heating (62.5 pg each) and protein extract of UEA-I(+) region (T; 0.5 mm^2 equivalent). The Cy3-labeled proteins were subjected to western blotting analysis using anti-Cy3 antibody. The arrow indicates the bands corresponding to biotinylated UEA-I. **(D)** LMA analysis of UEA-I probes with or without biotinylation (*B*) and heating (*H*). The left panel shows the scan image (Gain 125) of the probes (1 ng each) prepared as in (B). The right panel shows the net intensity of representative lectins indicated in the left panel. **(E)** LMA analysis of non-labeled and biotinylated UEA-I after heating (*H*; 62.5 pg each) and protein extract of the UEA-I(+) region (0.5 mm^2 equivalent). The left panel shows the scan image (Gain 85) of the probes and protein extract prepared as in (C). The right panel shows the net intensity of representative lectins indicated in the left panel.

Figure S3. Evaluation of the effects of anti-CK5 antibody on LMA analysis. (A) Detection of biotinylated anti-CK5 antibody. Protein extracts prepared from CK5(+) tissue fragments (*T*; 0.5 mm^2 equivalent) by Method 3, and the indicated amount of biotinylated antibody, were subjected to SDS-PAGE, transferred to the same membrane, and detected with horseradish peroxidase-conjugated streptavidin. The arrow indicates the bands corresponding to the heavy chain (H) of biotinylated antibody. **(B)** Comparison of Cy3-labeling efficiency among anti-CK5 antibodies with or without biotinylation and heating. Non-labeled and biotinylated antibodies were treated with or without heating with citrate buffer and then subjected to labeling with Cy3-SE. The Cy3-labeled antibodies (1 ng each) were subjected to western blotting analysis using anti-Cy3 antibody.

The arrow indicates the bands corresponding to the heavy (H) and light (L) chains of biotinylated UEA-I. **(C)** Comparison of protein amounts among non-labeled and biotinylated anti-CK5 antibody after heating (62.5 pg each) and protein extract of CK5(+) region (*T*; 0.5 mm^2 equivalent). The Cy3-labeled proteins were subjected to western blotting analysis using anti-Cy3 antibody. The arrow indicates the bands corresponding to the heavy chain (*H*) of biotinylated antibody. **(D)** LMA analysis of anti-CK5 antibodies with or without biotinylation (*B*) and heating (*H*). The left panel shows the scan image (Gain 125) of the antibodies (1 ng each) prepared as in (B). The right panel shows the net intensity of representative lectins indicated in the left panel. **(E)** LMA analysis of non-labeled and biotinylated anti-CK5 antibody after heating (*H*; 62.5 pg each) and protein extract of CK5(+) region (0.5 mm^2 equivalent). The left panel shows the scan image (Gain 95) of the probes and protein extract prepared as in (c). The right panel shows the net intensity of representative lectins indicated in the left panel.

Figure S4. Differential glycomic profiling of tissue fragments obtained from hematoxylin- and UEA-I-stained sections. Serial thymic sections were stained with hematoxylin or with UEA-I followed by hematoxylin staining with/without antigen retrieval, and subsequently subjected to LMD-LMA analysis. **(A)** Whole section images before/after tissue dissection. Numbers of dissected areas (1 mm^2 each) correspond to those of glycomic profile data presented in Table S3. **(B)** Fluorescent image of the whole section stained with UEA-I and hematoxylin. **(C)** Representative images of tissue dissection from the same area of each section. For the UEA-I/hematoxylin-stained section, the fluorescent image and bright-field image are also shown. **(D)** Western blotting analysis of protein extracts (0.05 mm^2 equivalent, N = 4 each). Total proteins after Cy3 labeling were detected using anti-Cy3 antibody (left panel) and semi-quantified by densitometric analysis of the image ranging from 25 to 100 kDa (right panel). Asterisks indicate

significant differences between samples (Tukey's honestly significant difference test, $P < 0.05$). **(E)** PCA score plot of glycomic profiles of the tissue fragments obtained from each section (0.5 mm^2 equivalent; N = 4 each). Glycomic profile data used for this analysis are presented in Table S3.

Figure S5. Tissue sections used for differential glycomic profiling of tissue fragments obtained from medulla/UEA-I(+) and cortex/UEA-I(-) regions by each method. Serial thymic sections were stained with hematoxylin or with UEA-I and then subjected to LMD-LMA analysis by Methods 1, 2, and 3. **(A)** Whole section images before/after tissue dissection. Numbers of the dissected areas (0.5 mm^2 each) correspond to those of the glycomic profile data presented in Table S4. **(B)** Fluorescent image of the whole section stained with UEA-I, which was used for analysis by Method 3.

Figure S6. Differential glycomic profiling of tissue fragments obtained from stained and unstained regions with the three probes. Serial thymic sections were stained for UEA-I, PNA, and CK5 and then subjected to LMD-LMA analysis by Method 3. To clarify the fluorescent staining, hematoxylin staining was additionally performed following CK5 staining. **(A)** Fluorescent images of whole sections stained with the three probes. The right panels show enlarged images of the indicated areas in the left panels. In the right panels, dashed lines indicate the border between the medulla (M) and cortex (C). **(B)** Whole section images before/after tissue dissection. Numbers of dissected areas (0.5 mm^2 each) correspond to those of the glycomic profile data presented in Table S5. **(C)** PCA score plot (left panel) and loading plot (right panel) of glycomic profiles of the tissue fragments corresponding to the medullary regions. Glycomic profile data (0.5 mm^2 equivalent; N = 3 each) used for these analyses are presented in Table S5.

(D–F) Glycomic profiles obtained from the medullary (black bars) and cortex (gray bars) regions determined from the staining patterns of UEA-I (D), PNA (E), and CK5 (F). Data are presented as the mean ± S.D. (N = 3). Asterisks indicate significant differences between samples (Student's *t*-test, $P < 0.05$).

Figure S7. Representative images of double fluorescent staining for UEA-I and DBA (A), DBA and CK5 (B), and PNA and DBA (C). Arrowheads indicate double-positive cells shown as white in the merged images. Dashed lines indicate the border between the medulla (*M*) and cortex (*C*). Insets show enlarged images of the indicated areas. Scale bars, 100 μm.

Figure S1

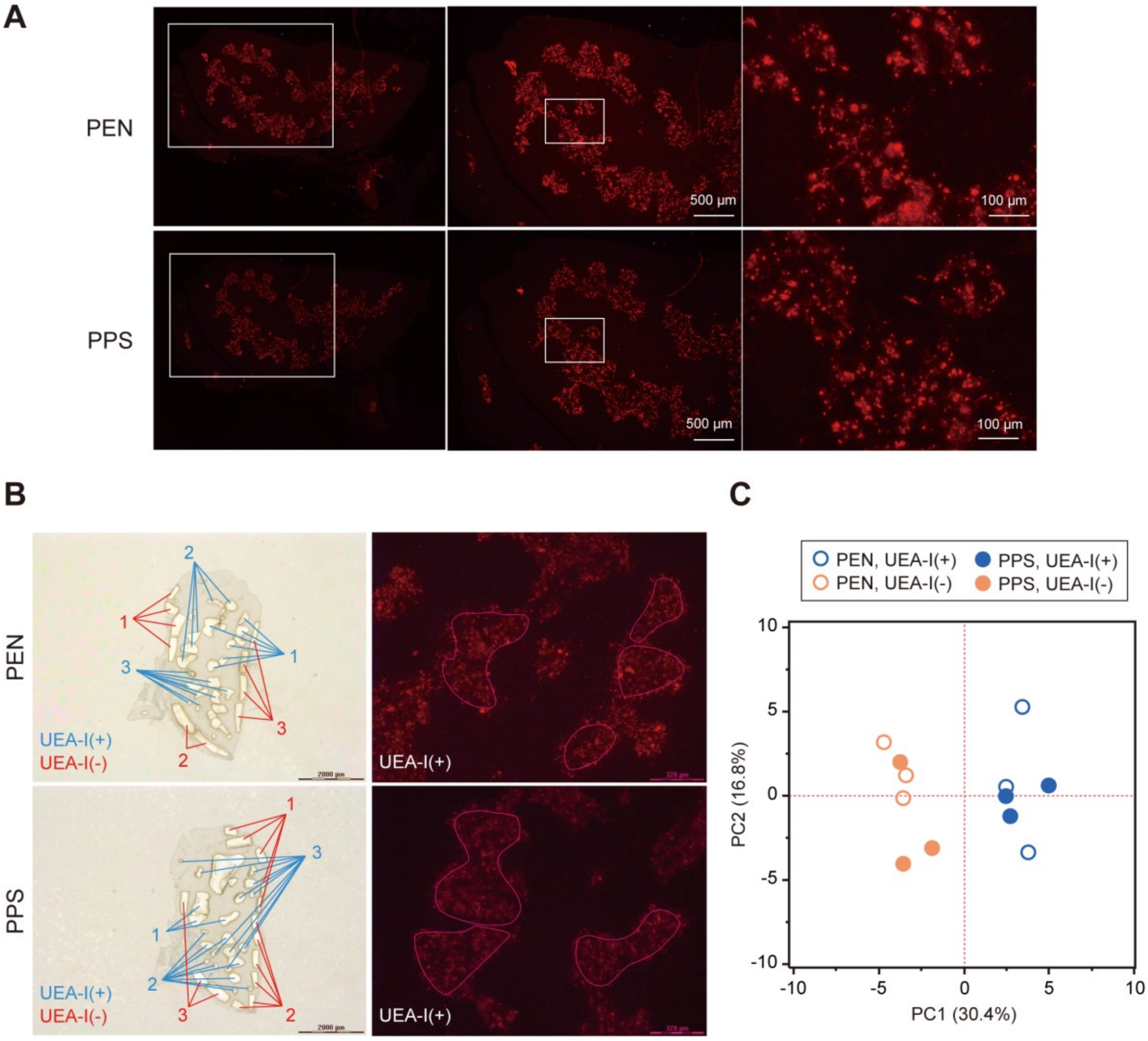
A
PEN
PPS
500 µm
100 µm
500 µm
100 µm
B
PEN
PPS
UEA-I(+)
UEA-I(-)
UEA-I(+)
UEA-I(+)
UEA-I(-)
UEA-I(+)
C
PEN, UEA-I(+)
PPS, UEA-I(+)
PEN, UEA-I(-)
PPS, UEA-I(-)
PC2 (16.8%)
PC1 (30.4%)

Figure S2

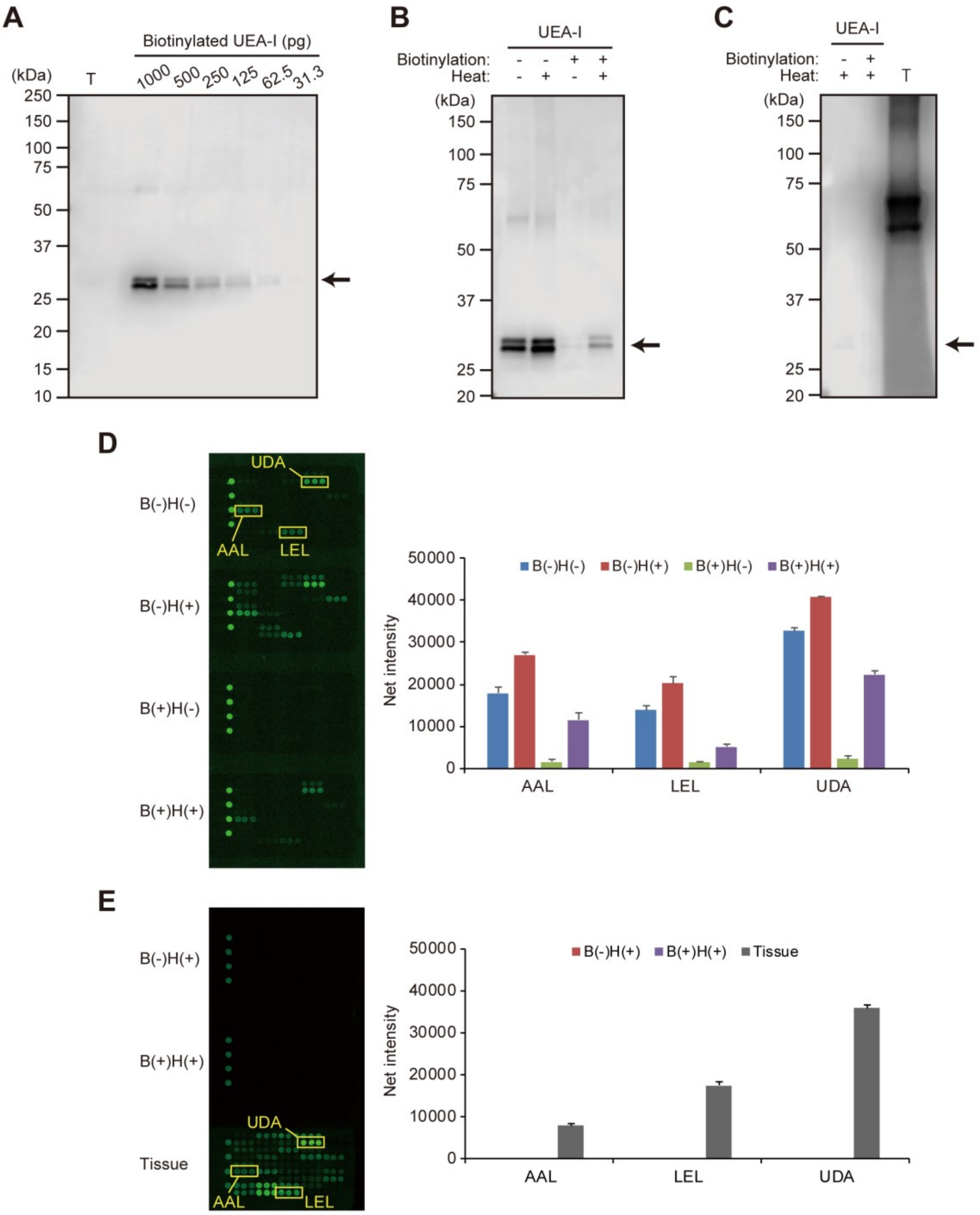
A
Biotinylated UEA-I (pg)
(kDa)
T
1000
500
250
125
62.5
31.3
250
150
100
75
50
37
25
20
15
10
B
UEA-I
Biotinylation: - - + +
Heat: - + - +
(kDa)
150
100
75
50
37
25
20
C
UEA-I
Biotinylation: - +
Heat: + +
T
(kDa)
150
100
75
50
37
25
20
D
UDA
B(-)H(-)
AAL
LEL
B(-)H(+)
B(+)H(-)
B(+)H(+)
Net intensity
B(-)H(-)
B(-)H(+)
B(+)H(-)
B(+)H(+)
50000
40000
30000
20000
10000
0
AAL
LEL
UDA
E
B(-)H(+)
B(+)H(+)
Tissue
UDA
AAL
LEL
Net intensity
B(-)H(+)
B(+)H(+)
Tissue
50000
40000
30000
20000
10000
0
AAL
LEL
UDA

Figure S3

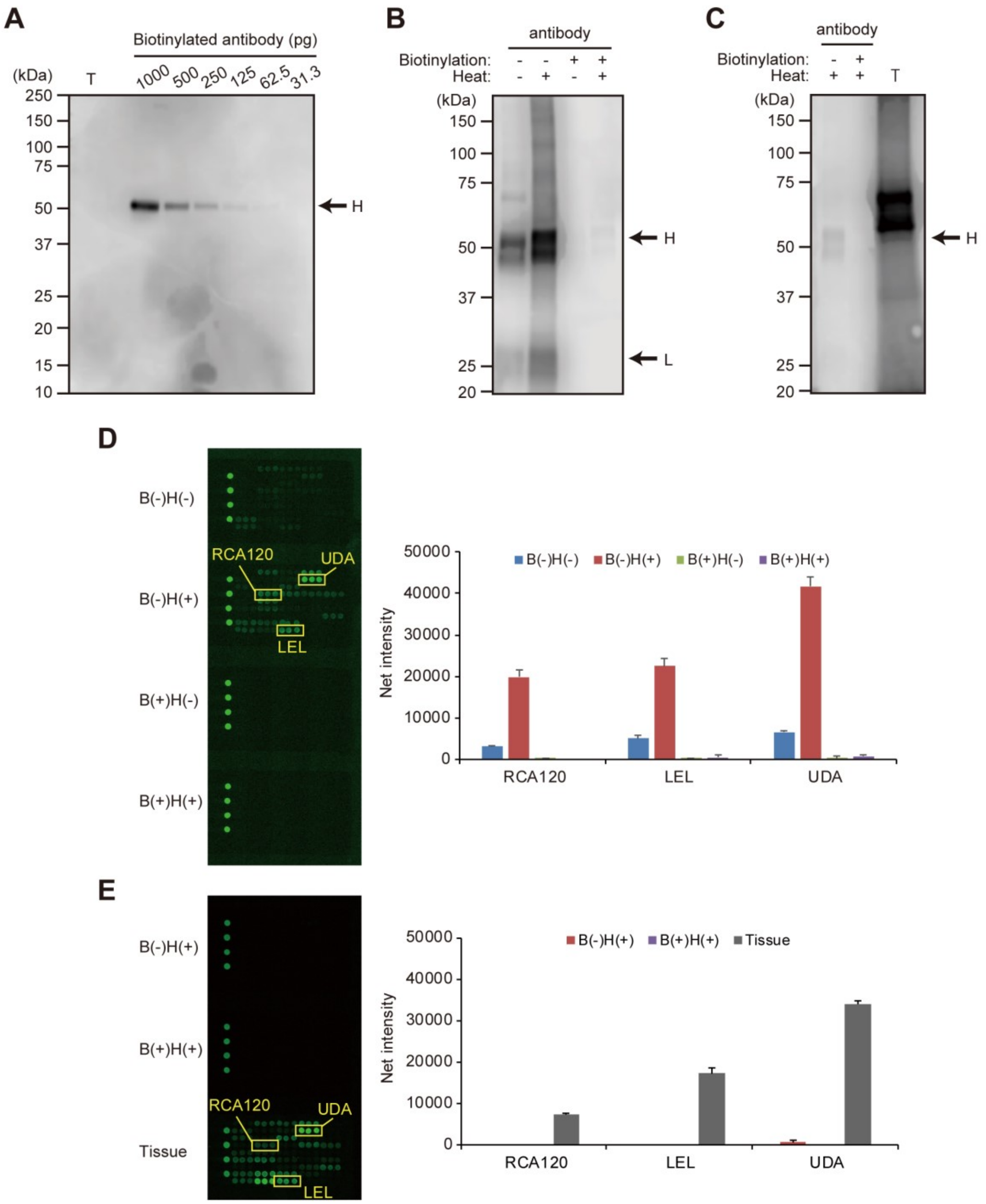
A
Biotinylated antibody (pg)
(kDa)
T
1000
500
250
125
62.5
31.3
H
B
antibody
Biotinylation:
Heat:
H
L
C
antibody
Biotinylation:
Heat:
T
H
D
B(-)H(-)
B(-)H(+)
B(+)H(-)
B(+)H(+)
RCA120
UDA
LEL
Net intensity
RCA120
LEL
UDA
E
B(-)H(+)
B(+)H(+)
Tissue
RCA120
UDA
LEL
Net intensity
RCA120
LEL
UDA

Figure S4

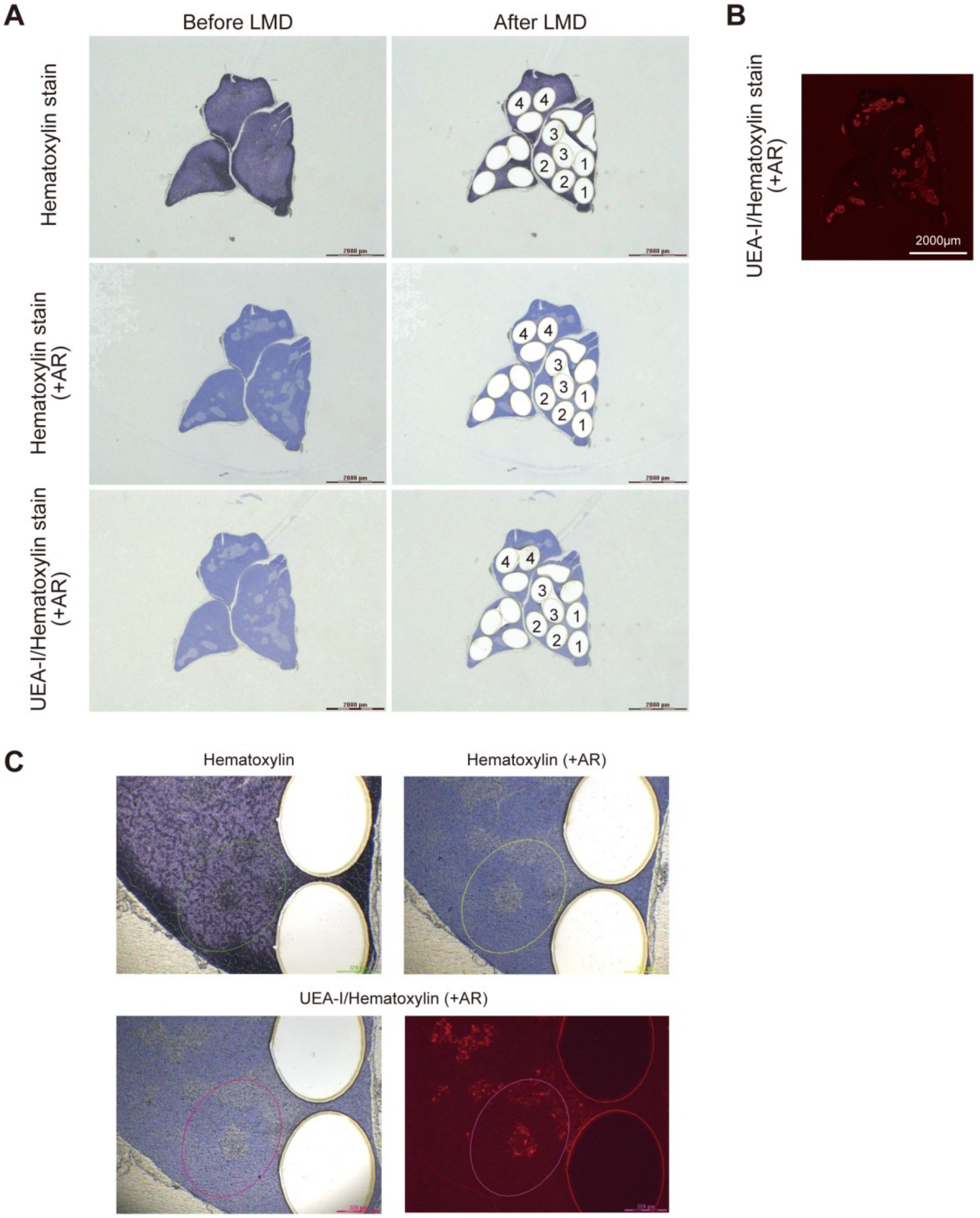

A
Before LMD
After LMD
Hematoxylin stain
Hematoxylin stain (+AR)
UEA-I/Hematoxylin stain (+AR)
B
UEA-I/Hematoxylin stain (+AR)
2000μm
C
Hematoxylin
Hematoxylin (+AR)
UEA-I/Hematoxylin (+AR)

Figure S4 (continued)

D

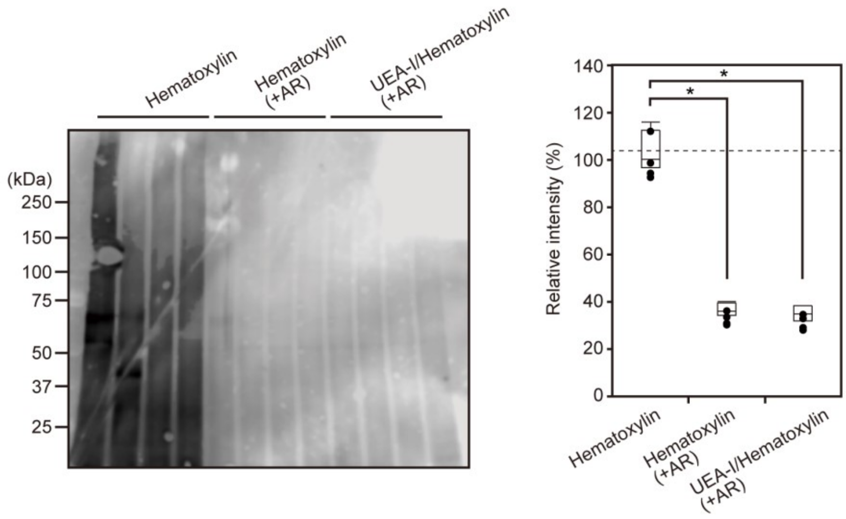

E

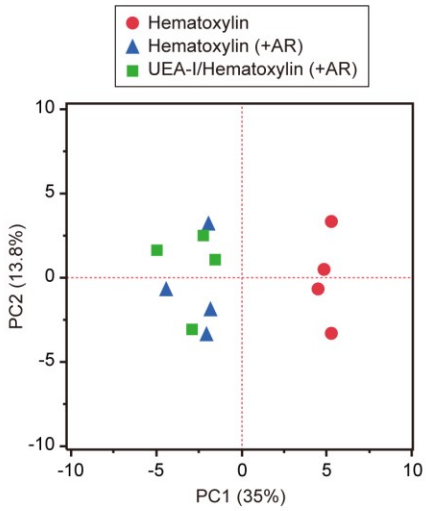

Figure S5

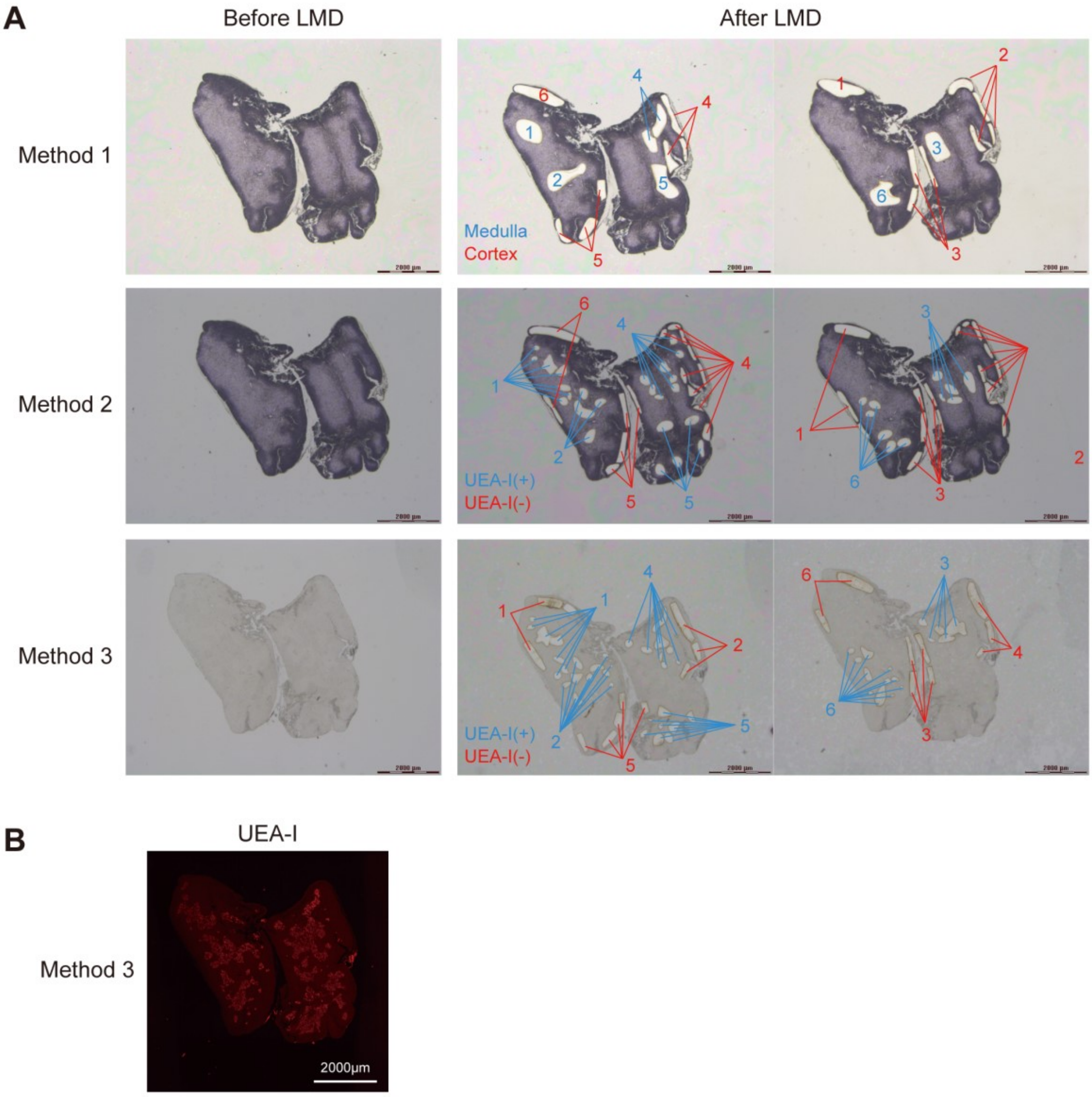
A
Before LMD
After LMD
Method 1
Medulla
Cortex
Method 2
UEA-I(+)
UEA-I(-)
Method 3
UEA-I(+)
UEA-I(-)
B
UEA-I
Method 3
2000μm

Figure S6

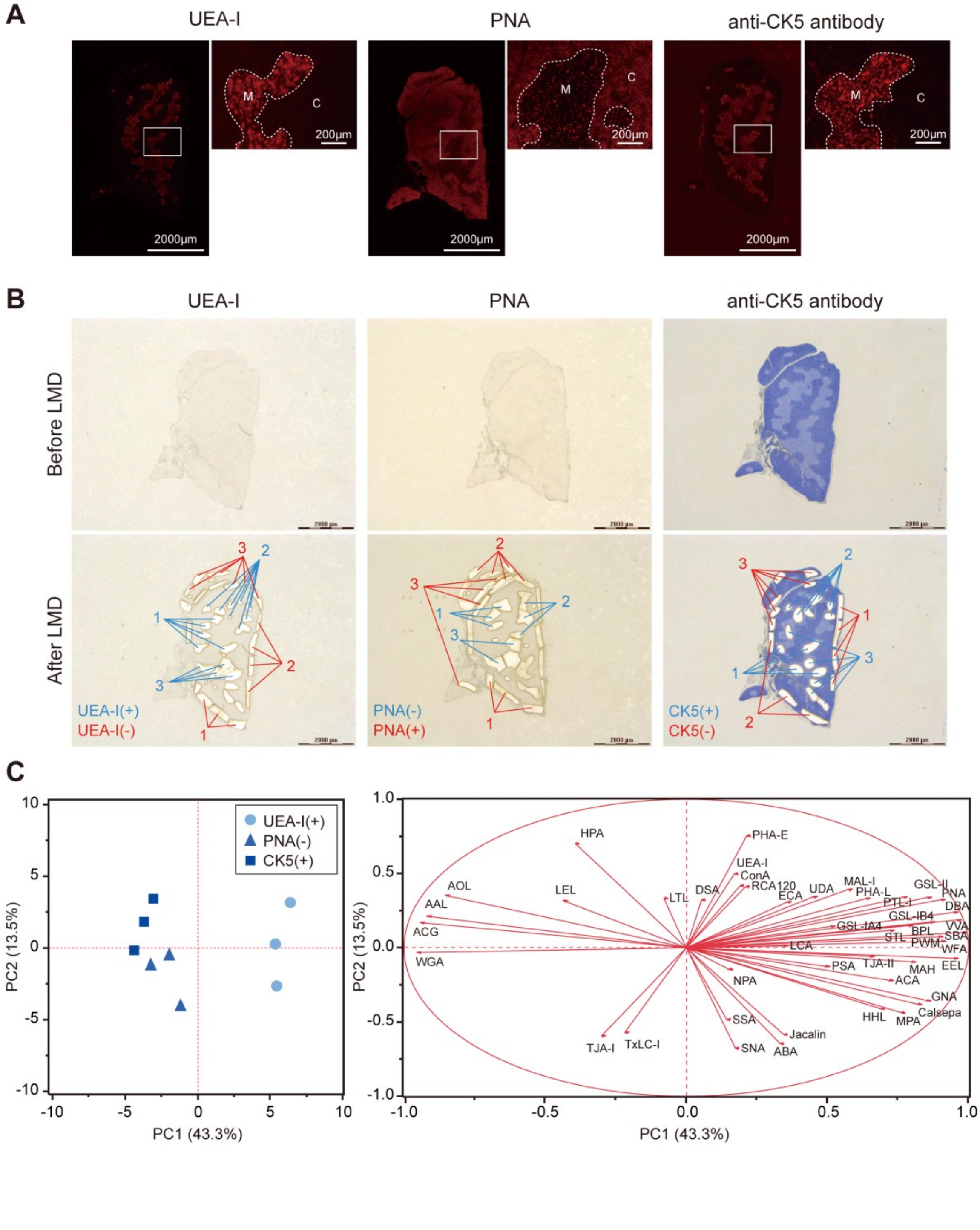

A
UEA-I
PNA
anti-CK5 antibody
M
C
200μm
2000μm
B
Before LMD
After LMD
UEA-I(+)
UEA-I(-)
PNA(-)
PNA(+)
CK5(+)
CK5(-)
C
UEA-I(+)
PNA(-)
CK5(+)
PC1 (43.3%)
PC2 (13.5%)
HPA
PHA-E
UEA-I
ConA
RCA120
DSA
LTL
LEL
AOL
AAL
ACG
WGA
ECA
UDA
MAL-I
PHA-L
PTL-I
GSL-II
PNA
DBA
GSL-IB4
GSL-IA4
BPL
STL
PWM
SBA
VVA
WFA
LCA
PSA
TJA-II
MAH
EEL
ACA
NPA
GNA
Calsepa
HHL
MPA
SSA
Jacalin
SNA
ABA
TJA-I
TxLC-I

Figure S6 (continued)

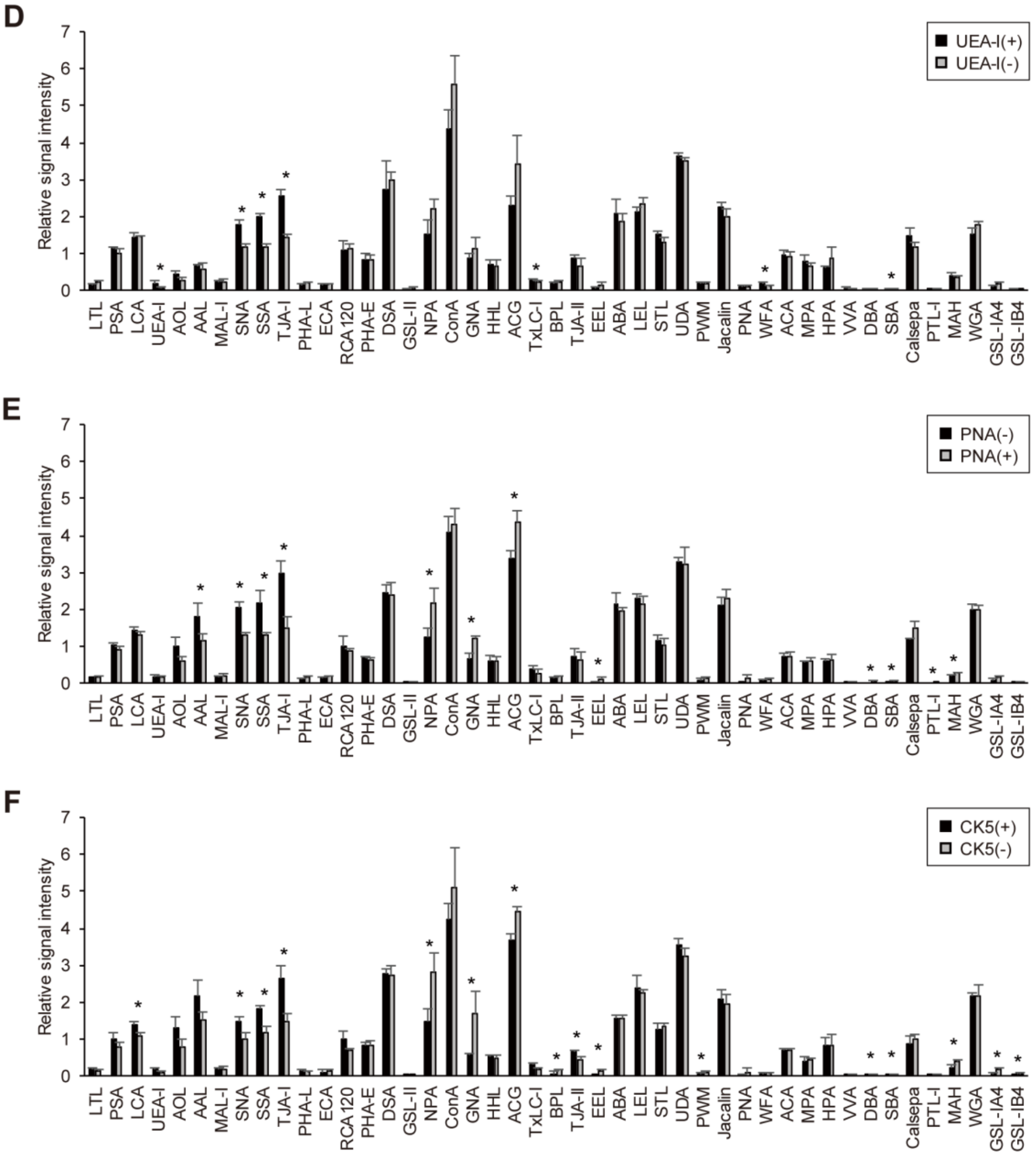

Figure S7

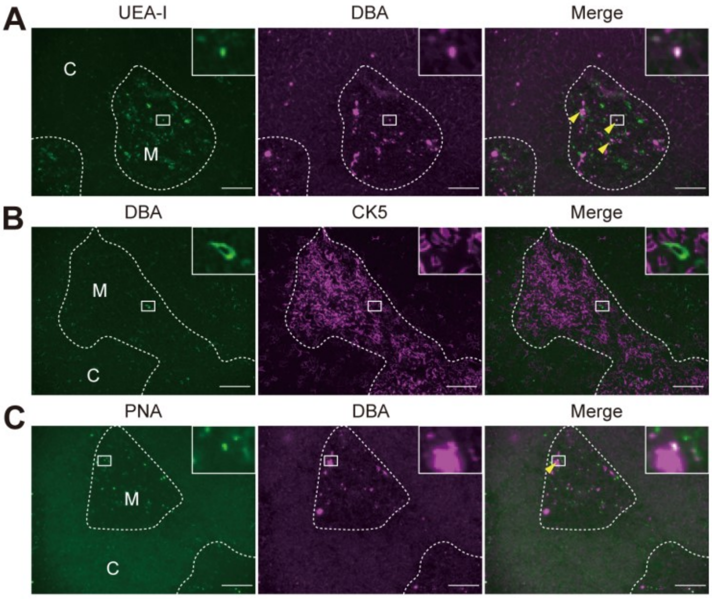

Supplement: Supplementary file 1 [file ijms-20-00700-s001.zip › Supplementary Figures and legends.pdf]
